# Integrating Computational Biology and Forward Genetics in *Drosophila*

**DOI:** 10.1371/journal.pgen.1000351

**Published:** 2009-01-23

**Authors:** Stein Aerts, Sven Vilain, Shu Hu, Leon-Charles Tranchevent, Roland Barriot, Jiekun Yan, Yves Moreau, Bassem A. Hassan, Xiao-Jiang Quan

**Affiliations:** 1 Laboratory of Neurogenetics, Department of Molecular and Developmental Genetics, Vlaams Instituut voor Biotechnologie, Leuven, Belgium; 2 Department of Human Genetics, Katholieke Universiteit Leuven School of Medicine, Leuven, Belgium; 3 Doctoral Program in Molecular and Developmental Genetics, Katholieke Universiteit Leuven School of Medicine, Leuven, Belgium; 4 Department of Electrical Engineering, Katholieke Universiteit Leuven, Leuven, Belgium; Stanford University Medical Center, United States of America

## Abstract

Genetic screens are powerful methods for the discovery of gene–phenotype associations. However, a systems biology approach to genetics must leverage the massive amount of “omics” data to enhance the power and speed of functional gene discovery *in vivo*. Thus far, few computational methods for gene function prediction have been rigorously tested for their performance on a genome-wide scale *in vivo*. In this work, we demonstrate that integrating genome-wide computational gene prioritization with large-scale genetic screening is a powerful tool for functional gene discovery. To discover genes involved in neural development in *Drosophila*, we extend our strategy for the prioritization of human candidate disease genes to functional prioritization in *Drosophila*. We then integrate this prioritization strategy with a large-scale genetic screen for interactors of the proneural transcription factor Atonal using genomic deficiencies and mutant and RNAi collections. Using the prioritized genes validated in our genetic screen, we describe a novel genetic interaction network for Atonal. Lastly, we prioritize the whole *Drosophila* genome and identify candidate gene associations for ten receptor-signaling pathways. This novel database of prioritized pathway candidates, as well as a web application for functional prioritization in *Drosophila*, called Endeavour-HighFly, and the Atonal network, are publicly available resources. A systems genetics approach that combines the power of computational predictions with *in vivo* genetic screens strongly enhances the process of gene function and gene–gene association discovery.

## Author Summary

Genome sequencing and annotation, combined with large-scale molecular experiments to query gene expression and molecular interactions, collectively known as Systems Biology, have resulted in an enormous wealth in biological databases. Yet, it remains a daunting task to use these data to decipher the rules that govern biological systems. One of the most trusted approaches in biology is genetic analysis because of its emphasis on gene function in living organisms. Genetics, however, proceeds slowly and unravels small-scale interactions. Turning genetics into an effective tool of Systems Biology requires harnessing the large-scale molecular data for the design and execution of genetic screens. In this work, we test the idea of exploiting a computational approach known as gene prioritization to pre-rank genes for the likelihood of their involvement in a process of interest. By carrying out a gene prioritization–supported genetic screen, we greatly enhance the speed and output of *in vivo* genetic screens without compromising their sensitivity. These results mean that future genetic screens can be custom-catered for any process of interest and carried out with a speed and efficiency that is comparable to other large-scale molecular experiments. We refer to this combined approach as Systems Genetics.

## Introduction

The demand by systems biology for *bona fide*, *in vivo* validated, biochemical interaction data and high quality functional annotations is much higher than the supply that geneticists are able to provide, principally because genetic approaches mainly focus on generating data on a gene-by-gene basis. On the other hand, computational predictions of gene function alone remain far from being accurate enough to be considered high-quality biological data. Integrated solutions, that combine the advantages of several approaches, should in theory provide both fast and physiologically relevant genetic data, while simultaneously increasing our understanding of biological processes. Genetic interactions in model organisms constitute a potentially invaluable source of *in vivo* interaction data for systems biology provided that throughput and speed can be increased. Currently, the number of known genetic interactions remains much smaller than the number of annotated physical interactions. For example, the BioGRID [1] database currently contains approximately 53,000 genetic interactions compared to almost 100,000 physical interactions.

Clearly, the power of genetic approaches is that they produce - by definition - data that is directly relevant in a living system. Genetic screens, either for specific phenotypes or for modifiers of gene function, are thus a valuable source of large-scale interaction data. However, the main disadvantage of large-scale genetic screens is that they are costly, labor intensive, and time consuming. Turning *in vivo* genetic screens into a staple of systems biology by making them easier and faster without compromising their accuracy would therefore represent a major advance.

In the bioinformatics community, process- or disease-related genes are, as of recently, being computationally predicted by taking advantage of the large amount of available sequence, function, annotation, and interaction data [2]–[13]. However to our knowledge, none of these methods have been used in combination with large-scale genetic experiments. Therefore, it remains unclear to what extent genome-wide, or even large-scale, computational predictions of gene-gene or gene-pathway associations, are biologically meaningful. Carrying out such screens on a large scale is difficult in human or mouse genetics, but the availability of genetic tools in *Drosophila melanogaster* together with collections of deficiency lines, mutants, and insertion lines, makes it an ideal model organism to investigate the concept of integrating genetic screens with gene prioritization methods.

Here, we integrate genetics and computational biology to identify genetic interactions underlying neural development in the *Drosophila* Peripheral Nervous System (PNS), a well-established model for neurogenesis. Proneural genes encoding proteins of the basic-helix-loop-helix (bHLH) super-family of transcription factors are essential for the initiation of neuronal lineage development in all species [14]–[18]. They act by forming heterodimers with the widely expressed bHLH E-proteins to bind a DNA motif called the E-box [19] and regulate the transcription of target genes. The highly conserved members of the Atonal (Ato) family are one example of proneural genes whose activity is required for the development of multiple lineages in vertebrates and invertebrates [14], [20]–[22]. Despite a solid understanding of when and where *ato*-like genes are required in the *Drosophila* PNS and how they interact with Notch signaling to select neural precursor cells (NPCs), the mechanisms that mediate their activity within NPCs and their specificity in inducing neuronal differentiation remain largely obscure.

To identify genes involved in *ato* mediated neural development we propose a strategy for functional gene prioritization in *Drosophila* called Endeavour-HighFly that uses the same data fusion method and user interface as the human gene prioritization method Endeavour [3],[23]. We identify 18 genes that interact with *ato* in two different contexts, including 2 previously uncharacterized genes, and use them to predict a core Ato interaction network. Furthermore, to broaden our strategy to other developmental processes, we prioritize the entire *Drosophila* genome for each of ten canonical biological pathways and generate a freely available database of candidate members or interactors for each pathway.

## Results

### Identifying Modifier Loci of *atonal*-Induced Neurogenesis in the *Drosophila* PNS

**Figure 1:**
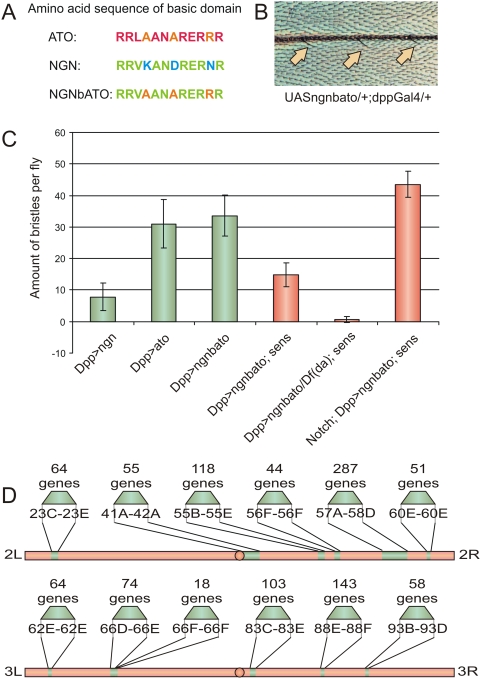
Overexpression of Ngn^bato^ in the *sens* mutant background provides a robust and sensitive phenotype for screening of *ato* dependent enhancers and suppressors. (A) Amino Acid sequence of the basic domain of Ngn (green) and Ato (red). The functionally critical amino acids are shown in separate colors. (B) Bristle phenotype on the third wing vein induced by Ngn^bato^ driven by dpp-Gal4. (C) Quantitative assay of ectopic bristle formation induced by Ngn^bato^ in wild type, *sens/+*, *da/+*;*sens/+* and *N/+*; *sens/+* backgrounds. Ato and Ngn are shown as positive and negative controls, respectively. Removing one copy of senseless reduces the amount of ectopic bristles. Removing one copy of *da* in a *sens/+* background results in a suppression of the phenotype, whereas removing one copy of *N* results in an enhancement of the phenotype. (D) Cytological position of the deficiency regions and amount of genes found within each atonal positive deficiency region on chromosome 2 and chromosome 3.

Three amino-acids within the basic domain of the first helix have been shown to mediate the specificity of *ato* function [24], and the same motif enables specific transcriptional activation of the nicotinic acetylcholine receptor beta-3 subunit by the *ato* orthologue Ath5 [25]. Substituting the same amino acids in the Ato-related mouse proneural protein Neurogenin 1 (Ngn1) for Ato group-specific residues (Ngn^bAto^) allows Ngn1 to induce neurogenesis in *Drosophila*. This induction mimics that caused by Ato itself and depends on the fly E-protein Daughterless (Da) and the proneural co-factor Senseless (Sens). Also, like endogenous proneural activity, it is antagonized by the Notch signaling pathway. Expression of the “Atonalized” form of mouse Ngn1, Ngn^bATO^ (Figure 1A) under the control of *dpp-Gal4* induces an average of ∼30 ectopic sensory bristles on the adult wing vein (n = 30; Figure 1B, C). This is in contrast to an average of only ∼7 bristles induced by Ngn1 itself (n = 26; p<0.001), but is similar to the number induced by Ato (n = 26, n.s.; Figure 1C). However, unlike Ato, Ngn^bATO^ induces significantly less lethality and many fewer wing deformities making it much easier to use in a large scale, quantitative genetic screen. In addition, just like for Ato, removal of one copy of *sens* reduces the number of Ngn^bATO^-induced bristles by 55.6% (Figure 1C). In order to bring the screen to a dosage critical value, a heterozygous *sens* mutant was introduced into the background of *UAS::Ngn^bATO^*; *dpp-Gal4*. The number of ectopic bristles with this system provides a sensitized and quantitative read out in which to screen for modifiers of Ato function.

To test the feasibility of isolating dominant modifiers of the number of ectopic bristles, we crossed *UAS::Ngn^bato^/Cyo*;*sens*,*dpp-Gal4/TM6c*, flies to *da* or *Notch* mutant flies. We find that removal of a single copy of *da* almost completely suppressed Ngn^bATO^ induced bristle formation (average of 0.7±0.9 bristles; n = 27, p<0.001), while removal of one copy of Notch strongly enhanced the phenotype (average of 43.5±4.1 bristles; n = 23, p = 0.002; Figure 1C). All together, these data suggest that the assay is both robust and sensitive and should enable the identification of specific quantitative modifiers involved in *ato*-dependent neurogenesis in the *Drosophila* PNS.

Following this strategy, a deficiency screen of the second and the third chromosomes for modifiers of *Ngn^bato^* misexpression was performed. The deficiency kit is a collection of fly stocks that each carries a deficiency, or deletion, chromosome uncovering multiple genes. The different deficiencies encompass most of the chromosome and deficiency screening is an established and rapid assay to identify chromosomal regions with enhancer and suppressor loci for a given phenotype or pathway [26]. To identify chromosomal loci that influence *ato*-induced neural development, 180 deficiency fly lines were crossed to *UAS::Ngn^bato^/Cyo*;*sens*,*dpp-Gal4/TM6c*, flies. Loci were considered positive if they altered the number of ectopic bristles on the adult wing vein by more than 30% compared to the number of bristles induced in sibling control flies, as well as in wild type Canton S flies, and if the change in bristle number was strongly statistically significant (p<0.01). Following these stringent criteria, 17 positive regions on chromosome 2 and 14 positive regions on chromosome 3 were identified. Since induction of ectopic bristles is a common property of all proneural genes, the identified loci might be involved in both *achaete-scute* and *ato* dependent neurogenesis. In order to identify Ato-specific loci, the individual candidate deletion stocks were tested with flies expressing *UAS::ato*, *UAS::ngn1*, and *UAS::sc*, respectively, under the control of *dpp-Gal4*. The loci which modified Ato misexpression, but not that of Sc or Ngn1 were considered to be Ato-specific loci. Of the 31 loci identified in the primary screen, only one failed to interact with any of the genes in the secondary screen. We find that 15 of the 31 loci interact with both *ato* and at least one other proneural gene, while 2 loci interact only with *ngn1* and 1 locus interacts only with *sens* (data not shown). The remaining 12 loci (6 on chromosome 2 and 6 on chromosome 3) interact specifically with *ato*. Examining the breakpoints of the overlapping deletions uncovering these 12 loci shows that they harbor 1056 annotated genes (Figure 1D and Table S1). Each of these loci is expected to harbor one or more *ato*-interacting genes.

The identification of the individual modifier genes from these regions is similar to the problem in human genetics where for a given human phenotype and its underlying chromosomal locus, identified by cytogenetic studies or linkage mapping for example, the individual disease-causing gene(s) need(s) to be identified. Besides directly providing interaction candidates, the twelve positive regions resulting from the deficiency screen provide an excellent opportunity to test the principle of gene prioritization on a large scale and in an unbiased setup. First we present a redesign of an existing gene prioritization approach that is specifically tuned towards the *Drosophila* genome, and then we use it to select the most promising candidates from the 1056 genes within the twelve positive regions.

### Endeavour-HighFly: A Tool to Prioritize *Drosophila* Genes through Genomic Data Fusion

**Figure 2:**
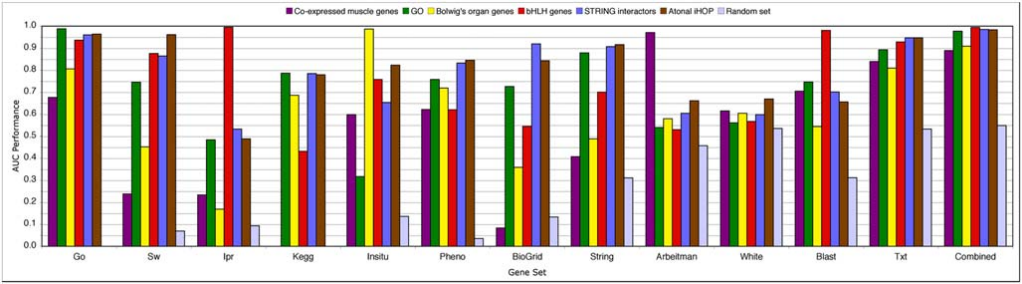
HighFly cross-validation results. The performance values, measured as Area Under the ROC Curve (AUC), obtained for all individual data sources (on the x-axis) are shown for several validation sets (each validation set is plotted in a different color; see legend). The AUC values for the overall prioritization, obtained by integrating all individual rankings, are also shown. Go: Gene Ontology; Sw: SwissProt keywords; Ipr: InterPro protein domains; Kegg: pathway database; Insitu: BDGP *in situ* hybridization data; Pheno: FlyBase mutant phenotypes; BioGrid: genetic and protein-protein interactions; String: protein-protein associations from STRING; Arbeitman: microarray data [39]; White: microarray data [40]; Blast: sequence similarity; Txt: Text-mining PubMed abstracts; Combined: or fused ranking by order statistics. Genes that are functionally related (e.g., same GO annotation or co-occurrence in abstracts) are prioritized well with the GO and text submodels, but also with the STRING and BioGRID submodels. Similarly, prioritization of genetically interacting genes works well with the BioGRID, STRING, GO and Text submodels. Genes that share similar microarray expression profiles or similar *in situ* expression patterns are prioritized well with their respective submodels. Lastly, genes that share similar protein domains are prioritized best by the InterPro and BLAST submodels.

To prioritize *Drosophila* genes we upgraded the existing Endeavour tool for gene prioritization [3],[23] by including *Drosophila* data sources (Table 1 and Materials and Methods) and we name this version Endeavour-HighFly, or HighFly for short. To test the performance of each individual *Drosophila* data source we carried out leave-one-out cross-validations (LOOCV; see Experimental Procedures) on several gene sets. Each set contains genes that are “similar” to each other for different reasons, for example genes with similar expression patterns or genes from the same pathway. We tested whether HighFly could identify the correct members of each set by leaving out one gene at a time and calculating the similarity between the left-out gene and the rest of the set. We found that HighFly ranks highly the left-out genes when at least one data source holds the information that this gene is related to the remainder of the gene set (for example, the expression data source is informative for the expression-related gene set) (Figure 2). Importantly, regardless of which particular data sources show the strongest performances, the performance of the combined or fused ranking (last column in Figure 2) is highly robust for all sets, it is not influenced by non-informative data sources, and it is almost always greater than 90% compared to a performance of ∼50% for randomly assembled sets of genes (Figure 2). These results validate the technical aspects of the implementation and suggest that HighFly performs robust prioritizations on *Drosophila* data sources.

**Table 1:** HighFly data sources.

Data type	Data source	Training	Scoring
Functional annotation	Gene Ontology [27]	GO term over-representation	Fisher's omnibus
	PubMed abstract profiles	Text-mining using gene-reference relations from FlyBase; average term weight vector	Cosine similarity
	SwissProt keywords [43]	Term over-representation	Fisher's omnibus
	KEGG [44]	Pathway over-representation	Fisher's omnibus
Gene expression	Life cycle of *Drosophila* microarray data [39]	Collection of all the expression profiles of the training genes	Average of 50% best Pearson correlation
	Tissue-specific gene expression in *Drosophila* larvae [40]	Collection of all the expression profiles of the training genes	Average of 50% best Pearson correlation
	In situ expression [45]	FBbt term over-representation	Fisher's omnibus
Protein sequence	InterPro [43]	Domain over-representation	Fisher's omnibus
	BLAST [46]	Ad hoc BLAST database of training genes prot. seq.	Blast test seq. to ad hoc db; rank by e-value
Allele phenotypes	FlyBase records “phenotype manifest in” [27]	FBbt term over-representation	Fisher's omnibus
Genetic interactions and protein-protein interactions	BioGRID [1]	List of training genes and all their interactors	Overlap between the test gene plus its interactors and the training list
	STRING [29]	Idem BioGRID	Idem BioGRID

Highfly training and scoring strategies for each data source.

Next, we investigated whether HighFly would be capable of finding genes that interact *in vivo* with *ato*. A training set, called TRAIN_Ato1 was assembled with the following genes: *ato*, *Brd*, *rho*, *Takr86C*, *pnt*, *dpp*, *Egfr*, *da*, *wg*, *sens*, *chn*, and *sca*. Because different sizes and compositions of training sets are possible, we tested the suitability of this training set for ato-related gene prioritization, by performing two tests. First, we assessed the content of some of the trained submodels. The trained GO submodel for this set contains “peripheral nervous system development”, “cell fate specification”, “eye morphogenesis”, “sensory organ development”, etc. as highly over-represented terms (p value<10^−09^). The Text submodel contains stemmed terms like “cell fate”, “notch”, “egfr”, “disc”. The InterPro submodel has no highly over-represented domains, but “Basic helix-loop-helix dimerization region bHLH” is marginally over-represented (corrected p-value = 0.07). Secondly, we tested the homogeneity of TRAIN_Ato1, by subjecting it to LOOCV and obtained an AUC performance of 98.5%, suggesting that TRAIN_Ato1 is a coherent and internally consistent training set. To test the possibility of obtaining biologically meaningful prioritizations, we performed a pilot test by prioritizing the right arm of chromosome 3 (chr3R) using TRAIN_Ato1 and then divided all the genes on the list into three groups: the top 1/3, the middle 1/3, and the bottom 1/3. From each group the top 30 genes for which stocks with mutant alleles are available from the public stock centers were examined for their modification of *ato*'s proneural activity, using the same bristle induction assay described above. Four positive genes were found in the top group (*rn*, *Antp*, *gro*, and *pros*), none in the middle group, and none in the bottom group (Table 2 and Table S2). Although the power of this preliminary test is greatly limited due to the relatively small number of genes tested (90) and the variability of available alleles, we found these results sufficiently encouraging to proceed with HighFly prioritizations of all twelve modifier loci found in the deficiency screen. However, to further evaluate HighFly, we intentionally chose a less stringent threshold of further validating the top 30% of ranked genes so as to compare the rankings of positive and negative genes with a sufficiently large sample size at the end of the screen.

**Table 2:** Validation of the HighFly screen results.

Name	Flybase ID	Chromosome	Rank on test region	Rank ratio on test region	Rank on chromosome	Rank ratio on chromosome	Phenotype^a^	P-Value
*Antp*	FBgn0000095	chr3	44/3341	1.31%	67/6027	1.10%	−31.40%	<0.001
*gro*	FBgn0001139	chr3	58/3341	1.74%	84/6027	1.40%	−69.90%	<0.001
*pros*	FBgn0004595	chr3	33/3341	0.99%	48/6027	0.80%	dead	
*rn*	FBgn0003263	chr3	13/3341	0.39%	21/6027	0.40%	44.20%	<0.001
*cas*	FBgn0004878	chr3	1/103	0.97%	123/6027	2.00%	−33.60%	<0.001
*dom*	FBgn0020306	chr2	12/287	4.18%	413/5252	7.90%	−31.40%	<0.002
*Egfr*	FBgn0003731	chr2	1/287	0.35%	2/5252	0.03%	−51.30%	<0.001
*fj*	FBgn0000658	chr2	3/118	2.54%	63/5252	1.20%	−50.00%	<0.001
*lilli*	FBgn0041111	chr2	3/64	4.69%	245/5252	4.70%	−35.40%	<0.002
*mus209*	FBgn0005655	chr2	2/44	4.55%	484/5252	9.20%	−36.70%	<0.005
*ppan*	FBgn0010770	chr3	3/58	5.17%	642/6027	10.70%	−33.60%	<0.002
*sbb*	FBgn0010575	chr2	2/118	1.69%	207/5252	3.90%	−33.60%	<0.001
*shg*	FBgn0003391	chr2	2/287	0.70%	30/5252	0.60%	−66.40%	<0.001
*smg*	FBgn0016070	chr3	3/18	16.67%	279/6027	4.60%	−100%	<0.001
*toc*	FBgn0015600	chr2	1/64	1.56%	834/5252	15.90%	−33.60%	<0.001
*zip*	FBgn0005634	chr2	1/51	1.96%	123/5252	2.30%	−38.10%	<0.001

Validation of Highfly on prioritized 3R chromosome and on different prioritized deficiency regions.

a The average percentage change of the number of ectopic bristles, compared to wild type controls.

### Identification of Novel *ato* Interacting Genes through the Integration of Gene Prioritization and Functional Genetic Modifier Assays

**Figure 3:**
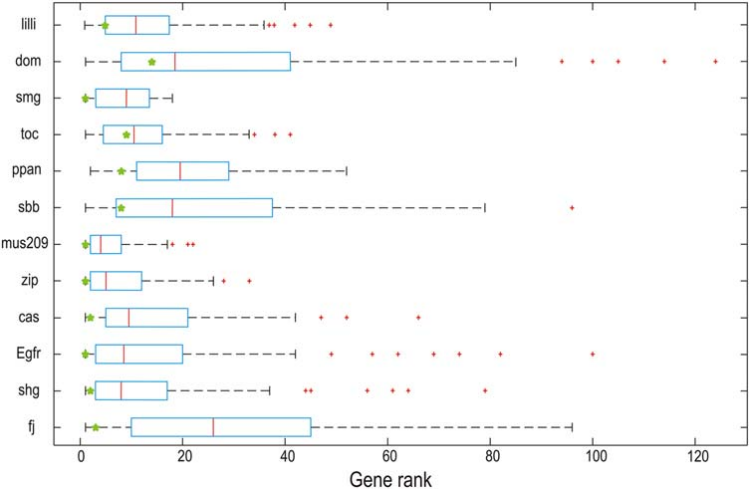
Ranking specificity of the *ato* interacting genes in the bristle assay. The observed rank of a positive gene, using the Atonal specific training set is compared to its rank obtained with a random training set (100 times). Shown is a boxplot of the 100 rankings of each positive gene using random training sets (y axis). The green asterisk represents the rank of the positive for the Atonal training set.

To identify candidate genes within the positive regions, all genes in each of the twelve positive regions were prioritized separately using TRAIN_ATO1 as training set and all 12 HighFly data sources (Table S3). For all genes that were ranked within the top 30%, a mutant stock, when available, was ordered from the public stock centers. Each mutant was then crossed to the sensitized tester fly stock (*uas::ngn^bato^/Cyo*;*sens*,*dpp-Gal4/TM6c*) and the bristles at the anterior-posterior margin (where *dpp-Gal4* is expressed) were counted and compared to the number of bristles observed in the control flies as described above. For twelve genes, namely *toc*, *lilli*, *Sbb*, *fj*, *mus209*, *zip*, *shg*, *Egfr*, *dom*, *smg*, *cas* and *ppan*, the number of bristles was significantly lower or higher (p<0.01) than in the control flies (Table 2, bottom panel). Each of these mutants were then tested against *uas::sc*; *uas::ngn1*, and *uas::ato* under the control of *dpp-Gal4* to check for the specificity of *ato* interaction. All of the genes modified only the *ato* gain of function phenotype (data not shown). We note that although mutants of genes that ranked in the top 30% of each locus were tested, 11 of the 12 ranked in the top 6% of their locus (Table 2 and Table S3), suggesting that HighFly prioritizations enrich strongly for positive interactions. Similar prioritizations were obtained by using a different high-quality training set (LOOCV AUC = 99.5%), assembled by selecting all 18 known interactors of *ato* from BioGRID (data not shown). In contrast when the same 12 genes were prioritized using 100 randomly assembled training sets, the median rank ratio was 0.247 compared to 0.02 for the *ato* training set (Figure 3).

An alternative analysis, instead of prioritizing each deficiency region separately, is to pool all candidate genes from the positive deficiency regions and prioritize this set in one analysis. We performed such a prioritization as post-analysis and found all 12 positives ranked in the top 10% (Table S1 and Figure S1). An examination of the contribution of individual data sources to the high rankings of the positive genes shows that for all positives, their high ranking is caused by high rankings for several data sources, rather than a single high ranking for one of the data sources (Figure S1), which supports our initial assumption of the added value of data integration for gene prioritization. In a second post-analysis, by comparing HighFly with existing online tools such as FlyBase [27], UCSC Gene Sorter [28], and STRING [29], we found that the use of a training set of genes related to *ato* is more favorable than a single gene query; and also that a gene ranking is more favorable for gene identification than a gene filtering (e.g., using a selection of Gene Ontology terms or a selection of FlyBase expression terms) (Text S1).

**Figure 4:**
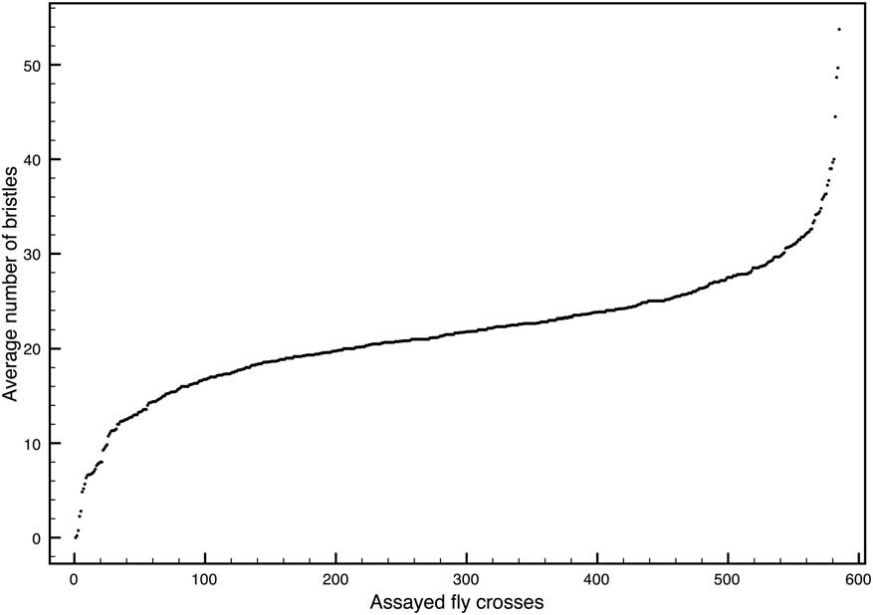
Distribution of the phenotypic range of the average number of bristles per genotype (n>10) plotted for all ∼600 assayed genotypes (y-axis). The shape of the curve conforms to the expectations for quantitative screens [31].

Functional inspection of the 16 positive genes (12 from the deficiency screen +4 from the pilot screen of 90 genes on chromosome 3R) by Gene Ontology statistics [30] revealed that this gene set is significantly enriched for developmental processes that require *ato* such as eye development and regulation of transcription (Table 3). Finally, we compared the phenotypic distribution of the effects of the modifier genes identified in our screen with the distribution documented for saturating forward genetic screens and cellular siRNA screens [31]. We find that despite the relatively small number of genes that need to be tested in a HighFly screen, the distribution of phenotypes mirrors that obtained in genome wide forward and reverse genetics screens (Figure 4). These data further support the power and accuracy of the integration of computational biology and genetics.

**Table 3:** Enrichment of GO-terms among the positive genes of the screen.

GO ID	Genes	Group count	Total count	P value	GO term
GO:0007444	*rn*; *pros*; *lilli*; *fj*; *ppan*; *EGFR*	6	259	0.000214	imaginal disc development
GO:0046530	*pros*; *lilli*; *sbb*; *EGFR*	4	72	0.000255	photoreceptor cell differentiation
GO:0016477	*zip*; *shg*; *dom*; *sbb*; *EGFR*	5	160	0.000276	cell migration
GO:0000904	*pros*; *lilli*; *shg*; *sbb*; *EGFR*	5	162	0.000284	cell differentiation
GO:0003700	*rn*; *cas*; *pros*; *lilli*; *Antp*; *sbb*	6	314	0.000447	transcription factor activity
GO:0007417	*cas*; *pros*; *shg*; *EGFR*	4	95	0.000622	central nervous system development
GO:0007420	*cas*; *shg*; *EGFR*	3	34	0.000693	brain development
GO:0007560	*rn*; *pros*; *lilli*; *fj*; *EGFR*	5	210	0.000764	imaginal disc morphogenesis
GO:0035218	*rn*; *fj*; *EGFR*	3	37	0.000811	leg disc development
GO:0001745	*pros*; *lilli*; *fj*; *EGFR*	4	108	0.000811	compound eye morphogenesis
GO:0007164	*fj*; *zip*; *EGFR*	3	38	0.000811	establishment of tissue polarity
GO:0000278	*zip*; *ppan*; *toc*; *mus209*; *EGFR*	5	223	0.000811	mitotic cell cycle

Selection of enriched GO-terms across the 16 positive genes. Full results table is available at http://med.kuleuven.be/cme-mg/lng/HighFly. All genes from chr2 and chr3 are used as background set.

**Figure 5:**
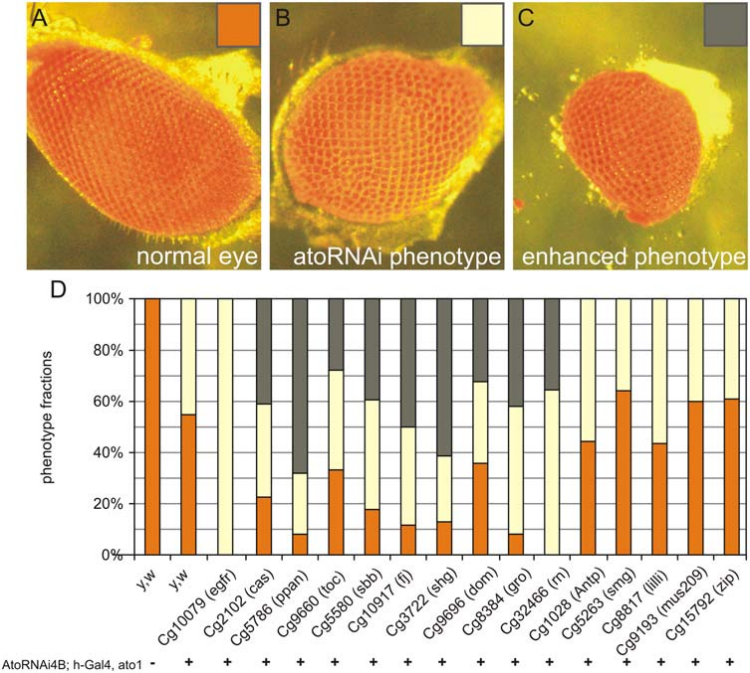
Effect of modifiers on eye size in atonal sensitized eyes. (A) Wild type eye with around 800 ommatidia. (B) effect from *ato-RNAi* on amount of ommatidia resulting in a population of flies with about 400 ommatidia per eye. (C) enhancement of *ato-RNAi* phenotype resulting in a population with smaller eyes with around 250 ommatidia per eye. (D) Overview of phenotypes observed when modifiers were crossed with atoRNAi flies, removal of one copy of the modifier results in a larger population of flies with smaller eyes for 9 of the previously identified modifiers. The other 5 modifiers did not show an alteration in eye phenotype, compared to the *ato-RNAi* phenotype in controls.

*ato* acts as a proneural gene for two different types of founder cells. The first is a subset of sense organ precursor (SOP) of the body wall and appendages and the second is the R8 founder cell of the retina. The major difference between the SOP and the R8 is that the SOP undergoes cell division to generate the sensory organ, whereas the R8 cell terminally differentiates. However, both cells share the property of recruiting neighboring cells into the *ato*-dependent fate; a property unique to *ato*, not shared by other proneural genes. We assessed whether genes identified in one context, also operate in the other. To this end, we tested the relationship between *ato* and its putative interactors in the developing fly retina, where *ato* function is well described [32]. In the retina, *ato* specifies the first photoreceptor, or R cell, the R8 (Figure S2A, B). The R8 then releases an *ato*-dependent EGF signal that organizes the rest of the retinal field and specifies the R1–R7. Loss of *ato* function in the retina results in the complete failure of retinal specification [33]. Expression of an *ato-RNAi* construct (A kind gift of A.P. Jarman) in the eye in *ato* heterozygous flies (*uas::ato-RNAi*;*h-Gal4*, *ato^1^*; see Materials and Methods) reduces R8 specification and consequently the recruitment of other R cells in a dose dependent fashion (Figure S2C,D). One copy of *ato-RNAi* produces a smaller eye with approximately half the normal number of ommatidia (Figure 5A,B). Mutants for the 16 genes identified in the screen were crossed to the *ato-RNAi* flies and scored for their ability to dominantly modify the *ato* RNAi phenotype. Ten of the 16 tested genes, namely *gro*, *rn*, *EGFR*, *cas*, *ppan*, *toc*, *sbb*, *fj*, *shg* and *dom* dominantly enhanced the *ato-RNAi* phenotype, with nine showing further reductions in eye size to approximately 250 ommatidia (Figure 5C, D). An 11^th^ gene, *pros*, was semi lethal. The remaining five genes, namely *Antp*, *smg*, *lilli*, *mus209*, and *zip*, did not appear to alter the *ato-RNAi* induced small eye size.

The data thus far suggest that at least 10 of the 16 genes we identified in the sensory bristle screen also interact with *ato* during retina development. Some of these genes such as *pros* are known for their role in neurogenesis [34], while the EGF receptor is well known for its close interactions with *ato* [35]. However, most of the genes we identified as genetic interactors of *ato* have not, to our knowledge, been previously shown to play a role in *ato*-dependent neurogenesis. Next we asked if these genes might be co-expressed with *ato* in the various PNS anlagen that derive from *ato* expressing precursors. We were able to obtain LacZ enhancer trap lines from stock centers for 10 of the 16 interacting genes (*dom*, *fj*, *lilli*, *mus209*, *pros*, *rn*, *sbb*, *shg*, *toc* and *zip*) to examine their expression patterns in the third instar larval (L3) imaginal discs. In the eye, antennal, leg and wing L3 discs, Ato marks the progenitor pools and the very early precursor cells of specific neuronal lineages. Senseless then marks the precursor cells during and after Ato expression. One enhancer trap, *rn*, did not show any obvious expression relationship to *ato*. Two of the 10 genes, *mus209* (fly PCNA) and *sbb* are generally expressed. An additional two lines, *toc* and *zip* showed expression in the posterior part of the eye disc (Figure S3A), suggesting a later function than that of *ato*. Finally, other five of the 10 tested enhancer traps showed a clear expression relationship with Ato (Figure S3). We observed strong lacZ expression in the L3 discs in Ato-expressing and Ato-dependent cells in the eye disc (*fj*, *lilli*, *shg*, *pros*), in the antennal Johnston organ precursor cells (*dom*, *shg*, *pros*), in the chordotonal organ precursor cells of the wing and leg imaginal disc (*dom*, *shg*, *pros*) (Figure S3 and data not shown). It should be noted that enhancer trap lines might reflect only part of the total expression pattern of the trapped gene.

### Identification of Uncharacterised *ato* Interacting Genes

The data above support the feasibility of rapidly and accurately identifying gene function through the fusion of *in silico* gene prioritization and *in vivo* genetic screens. One issue that faces all gene prioritization approaches is an expected bias towards genes with at a large amount of pre-existing information in several databases. Although this is still valuable in assigning novel functions to known genes, we reasoned that it would be interesting to test the performance of HighFly in the prioritization of genes about which there is little explicit information. Genes with limited annotations can potentially be ranked high due to data sources that are independent of existing knowledge, such as sequence similarity, protein domains, gene expression data, or protein-protein interaction data from high-throughput experiments. Indeed, 30 out of 96 genes, known only by their CG numbers, ranked in the top 10% of the *ato*-specific deletion loci identified in the initial bristle screen (Table S4). The recent availability of a genome-wide *in vivo Drosophila* RNAi library [36] allowed us to test these genes for their interaction with *ato*.

When no off-target effects were predicted, available RNAi lines were ordered and crossed to the *ato-RNAi* flies driven by the *h-Gal4* driver in an *ato* heterozygous background (*uas::ato-RNAi*;*h-Gal4*, *ato^1^*), as well as two different control lines; *h-Gal4*, *ato^1^* and *h-Gal4* alone. To avoid potential artifacts resulting from the RNAi approach, we set relatively stringent criteria: we searched for genes that show synthetic lethality specifically and only in combination with *ato-RNAi*, but show no phenotype under the two control conditions.

We were able to obtain a total of 36 RNAi lines for 24 uncharacterized genes ranking in the top 10% of positive deficiency regions. Eleven RNAi lines were lethal under all conditions and could not be evaluated further. The 25 remaining RNAi lines allowed us to perform knockdown of 17 genes. Of these, 2 genes (*CG1024*, *CG1218*,) caused lethality only in combination with *atoRNAi*, but not under control conditions (Table 4). As a further confirmation for the specificity of these interactions, we tested 51 RNAi lines for the bottom 10% ranking genes in each deficiency. None of these lines showed specific synthetic lethality in combination with *atoRNAi* (data not shown). Thus, the combination of HighFly prioritization, the RNAi library and genetic screening allows the rapid functional identification of previously uncharacterized genes.

**Table 4:** Synthetic lethal modifiers of *ato-RNAi* among the unknown genes.

Clone	CG number	Rank	CG-RNAi+atoRNA ;hGal4, ato^1^	CGRNAi+hGal4, ato^1^	CGRNAi+hGal4
*18597*	*CG1024*	7.7%	Lethal	No Effect	No Effect
*31685*	*CG1218*	6.6%	Lethal	No Effect	No Effect

Results of the phenotypes observed from RNAi screen.

### An Atonal Interaction Network

**Figure 6:**
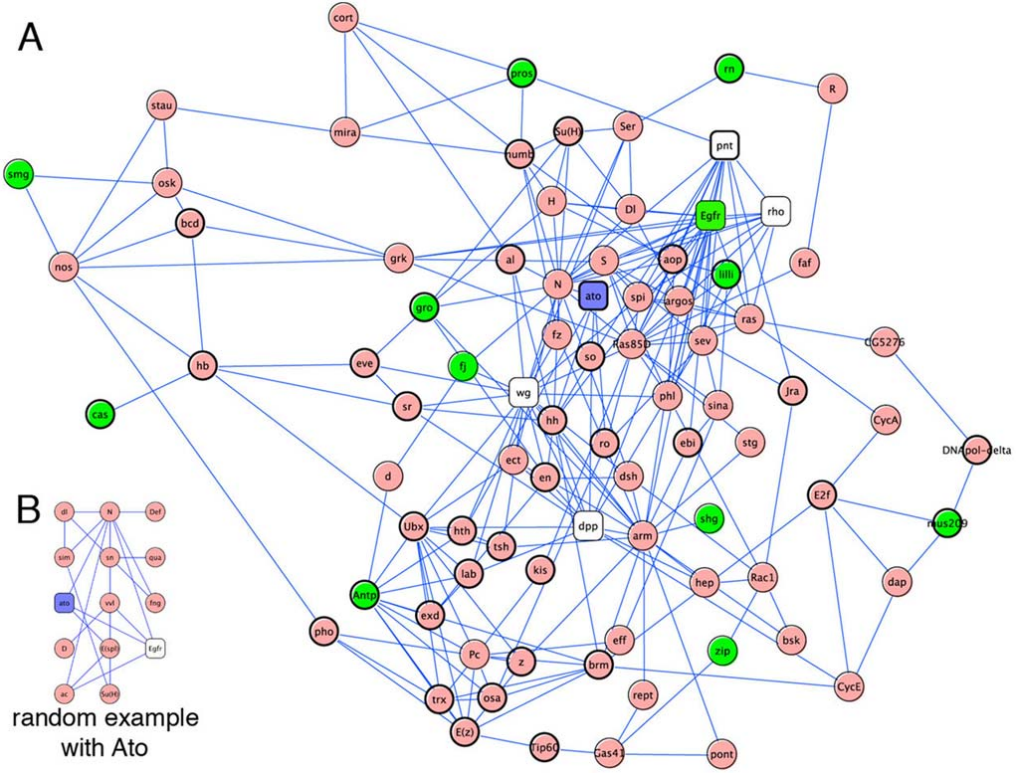
Protein-protein association subnetworks. The subgraphs are extracted from STRING connections with confidence score above 0.842, and aim to connect as many seed genes as possible. Seed genes may be connected through maximally two edges from either side. (A) The 16 positive ‘known’ genes are used as seed genes to validate their potential relationship. The resulting network is significantly larger and more interconnected than expected by chance and recovers Ato itself as member of the sub-network. (B) Example of a subgraph generated from a random selection of seed genes that does recover Ato. Only 29 out of 1000 random networks recover Ato by chance. These networks are significantly smaller than the network formed by the 16 genes from the screen. Green nodes are positive genes from our screen, while all other nodes are drawn from STRING interaction data (square nodes were part of the training set).

The combination of forward and reverse genetics tools and computational biology allowed the identification of 18, mostly novel, genetic interactions with the proneural gene *ato*. We sought to determine if the identified positive genes are functionally associated with each other, with *ato*, and with any of the other training genes that were used originally to identify these genes. To this end we used the STRING [29] protein-protein association predictions at 0.8 confidence level and determined the optimally connected sub-network that can be formed among the 18 positive genes, via maximally two other proteins (see Materials and Methods). We find that a network can be constructed that includes 12 of the 16 known genes (data not shown). As expected, the 2 unknown genes play no role in this analysis because of the lack of STRING data at this high confidence level. This analysis discovers Ato itself as member of the best network that connects the positive genes. We found that the maximal confidence level at which Ato is still part of the network is 0.842, and therefore used this stringency for further analyses. The network formed by the 16 known genes at this confidence level (Figure 6A) contains 84 nodes and 250 edges, and now includes 12 of the 16 positive genes and 6 training genes, including *ato*. *Egfr* is directly connected to ato; *fj*, *Antp* and *gro* are connected to *ato* via one other protein; *pros*, *rn*, *shg*, *lilli* are connected to *ato* via two other proteins and *cas*, *smg*, *zip*, *mus209* via three other proteins.

To determine the significance of finding a large interconnected network, which includes *ato*, starting from the 16 positive known genes, we generated 1000 random sets of 16 known genes. Specifically, we used only genes with a name in FlyBase and at least one GO biological process annotation. Only 29 of the 1000 networks contain *ato* and, on average, they contain 0.70 (S.D. = 1.13) training genes, 7.83 nodes (S.D. = 9.09), and 13.07 edges (S.D. = 19.28). An example of such a network is shown in Figure 6B. With a p-value of 0.029 to find Ato in the real network, p<0.001 to obtain 84 nodes, p<0.001 to obtain 250 edges, and p = 0.001 to recover 6 of the 11 training genes, we conclude that the positive genes we identified are strongly associated with each other and with Ato and its known interactors.

### A Database of Genome-Wide Gene Prioritizations in *Drosophila* for Ten Canonical Signalling Pathways

A particular feature of the HighFly tool is the speed of prioritization. We wondered whether this computational efficiency makes it possible to prioritize whole chromosomes or even the entire genome. To this end we asked if it is possible to rank the 16 known genes identified in our screen on their respective chromosomes, and if so, whether these rankings would be high. Table 2 shows the chromosomal rankings of these genes. All except one of the known genes rank within the top 10% of their respective chromosome (Table 2).

**Figure 7:**
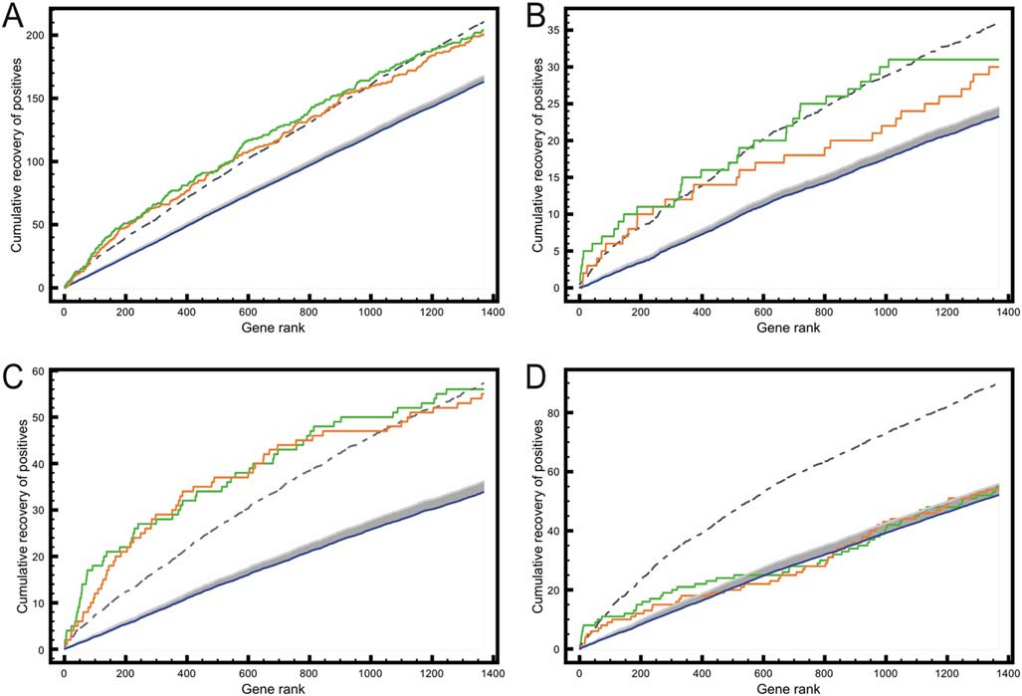
Comparison of whole-genome prioritizations for signaling pathways with results from RNAi screens obtained from http://www.flyrnai.org. For each of the four prioritizations the training set is based on Gene Ontology annotation for the respective pathway, namely GO:0000165 (MAPK) for the ERK pathway (A), GO:0007259 for JAK-STAT (B), GO:00016055 for Wnt (C), and GO:0007224 (smoothened) for hh (D). The green curve represents the cumulative recovery of positive genes when moving down the top 10% of the ranked gene list, using the full training set. The orange curve is similar to the green curve, but now excluding the known GO-annotated positives from the RNAi screen from the training set. The blue control curve is the average recovery curve of the positives, using 100 random training sets of known GO-annotated genes. The grey area represents a 95% confidence interval above the mean and the dotted curve represents two standard deviations above the mean, so that every point above the dotted curve represents a significant (p<0.05) enrichment of true positives. For three out of four, namely ERK, Wnt and JAK-STAT, a significant enrichment of positives is found at nearly all thresholds in the top 10% of the genome.

These data suggest that it is possible to obtain strongly meaningful gene prioritizations across large data sets. We sought to illustrate the general applicability of fly gene prioritization and simultaneously generate a second community-wide resource by prioritizing the entire genome to identify genes that are related to, or potentially involved in, either of ten signaling pathways, namely Transforming Growth Factor beta (TGFβ) receptor signaling pathway (GO:0007179), Epidermal Growth Factor Receptor (EGFR) signaling pathway (GO:0007173), Fibroblast Growth Factor Receptor (FGFR) signaling pathway (GO:0008543), Notch (N) signaling pathway (GO:0007219), Sevenless (Sev) signaling pathway (GO:0045500), Smoothened/Hedgehog (Smo/H) signaling pathway (GO:0007224), Toll signaling pathway (GO:0008063), Extracellular signal-Regulated Kinase (ERK; GO:0007259), JAK-STAT (GO:00016055) and Wnt signaling pathway (GO:0016055). To investigate the rankings in terms of biological processes we calculated GO over-representations for each top 100 ranked genes, excluding the training genes. We also excluded genes that were ranked in the top 100 for more than two pathways and GO-terms that were over-represented in more than four pathways. We find that typical overrepresented functions are cell adhesion and photoreceptor fate commitment for EGFR-related genes; cell migration for FGFR; neuroblast fate determination and equator specification for Notch; defense response for Toll; and ectoderm development for Wnt, suggesting that the prioritizations are biologically meaningful. Finally, we compared prioritizations for 4 of the 10 pathways- namely ERK, Wnt, Hh and JAK-STAT- for overlap with published genome-wide siRNA screens. We find significant overlap between the top 10% of the genome as prioritized by HighFly and the genes scored as positives in these screens for 3 of these pathways (Figure 7). Only the Hh pathway screen shows poor overlap with the prioritizations. Prioritizations and functional analyses, as well as the HighFly software, are available at http://med.kuleuven.be/cme-mg/lng/HighFly.

## Discussion

The molecular unraveling of biological processes in the post-genome era is characterized by the use of high-throughput experiments and the integration of prior knowledge (e.g., the use of GO-statistics to select microarray generated gene clusters), and is therefore supported and guided by bioinformatics. Genetic screens in model organisms such as *Drosophila melanogaster* are also high-throughput experiments, but they are yet to be aided by computational techniques, as an integral part of the screen itself. We sought to demonstrate the power of an integrated approach that combines high-throughput *in silico* and *in vivo* genetic approaches. This integration allowed us to quickly identify novel genetic interactions during neural development in the fly PNS, while significantly reducing the workload of the genetic screen. First, a classical deficiency modifier screen is performed. Then, instead of assaying all the genes located within the positive deficiency regions, the best candidates are selected computationally. This is done by integrating multiple heterogeneous genome-scale data sources, both representing published knowledge (e.g., functional gene annotations or protein-protein interactions), genome sequences, and experimental data (e.g., gene expression data or phenotypes). As such, we were able to assign novel functions for known genes whose involvement in *ato*-dependent neural development was unknown, as well as describe functions for uncharacterized genes.

A major advantage of genetic screens is that they are unbiased: they can reveal a function for a previously unknown gene. Although gene prioritization based on available data would have been expected to affect this property of screens, our data indicate that this is not necessarily the case. Even genes with very little explicit information, and no known function could be identified both as high ranking and as *bone fide* interactors *in vivo* in our HighFly supported screen. In addition, our data suggest that the combination of HighFly prioritizations and transgenic RNAi lines can result in very rapid functional gene discovery.

The use of an integrative screening strategy combining computational biology with medium or high-throughput screening assays is likely to be applicable to a broad range of screening assays (from *in vitro* to *in vivo* assays) beyond *Drosophila* genetics. Essentially any assay designed around evaluating a given gene, and for which whole-genome screening is outside the reach of the typical lab, could benefit from strategies similar to ours. Even with more extensive resources, it may be more productive (at equal time and cost) to evaluate several prioritized screens than a single whole-genome screen. Obviously, the strategy we propose is not applicable in the case where extremely little is known about the molecular basis of a phenotype (because of lack of a training set) while a genetic screen would still be feasible. It is a clear research challenge for computational biology to develop methods applicable to such a situation.

A further advantage of our integrated systems genetics approach is the combination of speed and accuracy of gene function discovery. In this work we tested a total of 180 deletion lines, 220 mutants and 36 RNAi lines to identify 18 *ato* interacting genes, representing a discovery rate of ∼5%. It should be noted that the 220 mutants tested include 90 mutants examined only for the purposes of testing the prioritizations as well as 78 mutants ranking between 10% and 30% of their deletion regions. Our data clearly indicate that testing genes ranked in the top 10% only will suffice to discover the vast majority of sought after genes: 17 of the 18 genes identified (∼94%) rank in the top 10% of their tested regions. Thus, assuming all genes have available RNAi lines or mutant alleles, testing only 96 genes, after the initial deficiency screen, would have identified at least 17 *ato* interacting genes, a discovery rate of almost 18%. In this regard we note that Endeavour-based prioritizations appear to outperform existing tools. We believe this to be due to three main properties namely the use of a multi-gene training set, the integration of multiple data sources, and the production of gene rankings.

The genes we find to interact with *ato* reveal an interaction network underlying early neural differentiation. Network analysis reveals two important aspects of the screen. Although neither Ato nor its known interactors were included in the query, the best network found includes Ato and almost all of its known interactors. In addition network analysis yields a number of interesting insights. First, most of the 89 genes in this network are signaling molecules and transcription factors belonging to the Notch, Wnt, EGFR, Dpp and Hh pathways. These pathways are known to interact with *ato* and our data suggest that the newly identified *ato* interacting genes may be members of these pathways or may implement the interactions between *ato* and these pathways. Second, most of the genes tested for both bristle formation and retinal development interact with *ato* in both assays. This suggests that *ato* may work with a core group of genes to implement context-specific neural fate decisions. One exception to this appears to be genes acting in cell division (*mus209*, *lilli*, *zip*) that, not surprisingly, interact in the bristle assay, but not the R8 assay. Third, we note that HighFly was able to predict the interaction of uncharacterized genes with *ato*, which network analysis alone, would have not been able to predict.

In summary, a systems genetics [37] approach not only identifies novel functions for individual genes with great speed and accuracy, but, as would be desirable in a systems biology context, also uncovers the structure and functional attributes of the network formed by these genes. Yet, the main advantage of systems genetics over other systems biology approaches is that the results are physiologically relevant by definition, because they are discovered directly *in vivo*.

The HighFly tool can perform prioritizations on the entire fly genome. We have done this for ten major signaling pathways, but many other prioritizations are possible, depending on the interest of the user. HighFly and its prioritizations are public resources that we hope will contribute to enhancing the speed and accuracy of functional gene discovery *in vivo* and establishing classical genetics as a fundamental tool of systems biology.

## Materials and Methods

### Fly Strains and Genetics

All crosses were performed at 25°C, except for the *atoRNAi* eye screen crosses which were performed at 28°C, on standard fly food. Deficiency kits, *LacZ* enhancer trap flies and all mutant lines were obtained from the Bloomington and Szeged stock centre. The *atoRNAi* lines were kindly provided by Andrew Jarman, and the RNAi lines for uncharacterized genes were obtained from the Vienna *Drosophila* RNAi Center (VDRC).

### Immunohistochemistry

Third instar larval imaginal discs were dissected in 1× PBS. Discs were fixed with 4% formaldehyde in 1× PBT for 15 minutes. Then, washed five times (15 min/T) in 1× PBT. Blocking and antibody incubation were performed as described [38]. The antibodies used were: sheep anti-ATO (1∶250), rabbit anti-GFP (1∶1000), rat anti-Elav (1∶100), guinea pig anti-SENS (1∶1000) mouse anti-βgal (1∶1000), rabbit anti–βgal (1∶1000). Secondary antibodies were always used 1 in 500. Samples were mounted in Vectashield mounting medium and detected using confocal microscopy (BioRad 1024, Hercules, California, United States and Leica DM-RXA, Wetzlar, Germany).

### Genetic Screen

The fly strain *w*; *UAS::ngnbato/CyO*; *sens*, *dpp-GAl4/TM6* was used to set up crosses with deficiency lines. The number of the ectopic bristles was used as a parameter to reflect the strength of the proneural function of Ato in this context [24]. When a deficiency region caused a significant change in the number of ectopic bristles, the corresponding deficiency line was further crossed to three fly lines *UAS::ato*; *dpp-Gal4*, *UAS::ngn*; *dpp-Gal4* and *UAS::sc*; *dpp-Gal4* and the number of ectopic bristles was counted. Deficiencies were considered *ato* specific when they altered the amount of bristles generated by *UAS::ato/cyo*; *dpp-Gal4/TM6*, and not by *UAS::ngn*, *dpp-Gal4/TM6* or *UAS::sc*, *dpp-Gal4/TM6*. Within these deficiency regions, high-ranking mutant lines available in the stock centre were ordered and crossed to *w*; *uas::Ngnbato/CyO*; *sens*, *dpp-GAl4/TM6*. If a mutant still caused a significant change in bristle number, the corresponding gene interacts with Ato. The positive genes were tested with flies expressing *UAS::ato*, *UAS::ngn1* and *UAS::sc* respectively under *dpp-Gal4* control to check for specificity. All ectopic bristles were counted under stereomicroscope. For all statistic analysis, the sample number is n = 10, and a significant difference between two average values is defined as p≤0.01. The eye phenotype screen was performed by crossing *w*; *UAS::atoRNAi/CyO*; *h-Gal4*, *ato^1^/TM6C*, which reduced the eye size in 50% of the flies, with the mutant strains identified in the bristle screen. Positive genes for retinal modifiers of *ato* were mutants that enhanced or suppressed the atoRNAi phenotype. RNAi strains were crossed to *h-Gal4*; *ato^1^/TM6* and *h-Gal4* as controls. Only the one showing synthetic lethality specifically with *w*; *UAS::atoRNAi/CyO*; *h-Gal4*, *ato^1^/TM6C*, but not with two controls was considered as positive.

### Gene Prioritization

The gene prioritization method [3],[23] works as follows. First, a set of training genes is defined to describe the particular process under study. For each data source, the following data for the training genes are assembled: (1) a gene's function derived from FlyBase GO annotation, textual information extracted from PubMed abstracts, SwissProt keywords and KEGG pathway membership; (2) a gene's expression pattern derived from two general *Drosophila* microarray data sets [39],[40] and embryonic *in situ* expression patterns from the Berkely Drosphila Genome Project (BDGP); (3) a gene's protein sequence from Ensembl and its protein domains from InterPro; (4) described mutant phenotypes from FlyBase; and (5) described genetic interactions or predicted protein-protein associations from BioGRID and STRING. The applied training and scoring strategies for each data source are described in Table 1. For each gene in a “test set” the similarity with a submodel is calculated and the ranks according to individual submodel scores are integrated using order statistics, yielding a q-value. The q-value is transformed into a p-value according to fitted distributions, depending on the number missing values. Finally, the test genes are ranked according to this p-value.

### Leave-One-Out Cross-Validation (LOOCV)

We assembled sets of genes involved in the same signaling pathway, tested on eight pathways defined by GO; genes with similar expression patterns using an expression cluster from Arbeitman et al. [39] and a second cluster of all genes expressed in Bolwig's organ from FlyBase; genes with the same protein domain, namely the bHLH domain; all genes that interact with the same gene, tested on all interactors with Atonal from BioGRID; and genes that are co-cited with a specific gene in PubMed abstracts, namely genes cited with *ato*, extracted using iHOP [41]. In LOOCV, every gene from every validation set is, in turn left out, and the ranking of the left-out gene within a set of 99 randomly selected genes is recorded. From all these rankings, Receiver Operating Characteristic (ROC) curves are generated and the area under this ROC curve is used as a measure of the performance of each individual data source and of the integrated prioritization.

### Network Extraction

The aim of the network extraction is to obtain a subgraph that connects the genes of interest (the seed genes). Network connections were extracted from the STRING protein-protein associations, using a minimum edge confidence (above 0.8). We define the connecting nodes (the non-seed genes) in the subgraph as the nodes that are on the shortest path(s) between two or more seed genes. To identify those connecting nodes, a multiple sources breadth-first search is performed, which is initialized with the seed genes. During the search, the minimum distance to the seed genes is recorded until seed genes are reachable from one another. Upon completion, the final network is obtained by exploring the shortest paths, starting from the seed genes, that have a maximum length of 4 and that connect at least two seed genes. Hence, the extracted network is made of one or more connected components and may not include all the seed genes. The obtained networks were visualized using Cytoscape [42].

## Supporting Information

Figure S1 Contributions of the HIGHFLY data sources to the overall ranking. One HIGHFLY prioritization was performed on all 1056 genes that are contained within the 12 positive ato-specific deficiency regions. The first column shows the rank of the positive genes for the overall ranking obtained by the fusion of all the individual sources (columns 2–13). Grey squares represent missing data for that particular gene and data source. The genes with no or limited existing knowledge, such as CG1218 and CG1024 can still be ranked high. CG1218 is ranked high because of similarities with the training set through BioGRID (CG1218 interacts with *sine oculis*), BLAST (CG1218 has sequence similarity with *chn*, E-value 18.2) and Microarray_2 (similarities between CG1218 and the training set according to microarray gene expression data). CG1024 has similarities with the training set through BLAST (CG1024 has sequence similarity with *senseless*, E-value 0.54), InterPro (CG1024 contains a Zinc finger motif, C2H2-type, like *senseless*), and Swissprot (CG1024 contains the keyword “Zinc-finger, DNA binding”).
(0.51 MB TIF)

Figure S2 Expression of *ato-RNAi* inhibits retinal differentiation. Eye discs are oriented with posterior located to the left. A) Scheme of *ato* dependant retinal induction using the formation of one photoreceptor cluster as example, first Ato is expressed in a stripe of cells, than, due to lateral inhibition *ato* expressing cells are restricted to three cells and then a single cell, the R8, which begin to express Sens. The other 7 photoreceptors are recruited in a reiterative way. When these neurons mature they express Elav. B) wild type control eye disc stained for Elav (blue), Sens (green) and Ato (red). C, D) Expression of *ato-RNAi* causes dose-dependent loss of retinal differentiation with one copy (C) leading to the appearance of gaps in the Elav pattern, and two copies (D) leading to a major failure of photoreceptor differentiation.
(0.93 MB PDF)

Figure S3 Overview of the expression pattern of *ato* interacting genes detected using *LacZ* enhancer trap lines or antibodies. (A) Overview of eye-antenna imaginal disc, with posterior to the right, ed: eye disc, ad: antenna disc. (A′) β-gal staining of *LacZ* enhancer trap flies mimicking the expression pattern of the genes nearby. Enhancer traps of *zip*, *fj*, *sbb*, *shg*, *toc*, and *lilli* show expression patterns in the eye disc. (B) eye disc of *lilli* enhancer trap flies, showing co-localization between Ato (B″) Sens (B′) and β-gal (B′). (C) Eye disc of *fj* enhancer trap flies, showing co-localization between Ato (C″) Sens (C′) and β-gal (C′). (D) Leg disc of *shg* enhancer trap flies, showing co-localization between Ato (D″) Sens (D′) and β-gal (D′) in the leg chordotonal organ precursor. (E) Wing disc of *dom* enhancer trap flies, showing co-localization between Ato (E″) Sens (E′) and β-gal (E′) in the wing chordotonal organ precursor. (F) Antennal disc of *dom* enhancer trap flies, showing co-localization between Ato (F″) Sens (F′) and β-gal (F′) in the Johnston organ precursor.
(3.02 MB PDF)

Table S1 Results of the prioritization of all 1056 genes from the 12 positive deficiency regions together, indicating the result of the bristle assay. Negatives and positives are indicated with orange and green shading respectively.
(0.38 MB XLS)

Table S2 Results of the prioritization of chromosome 3R. The first 30 available mutant stocks are shown for the top 1/3, middle 1/3, and bottom 1/3 of chromosome 3R after prioritization. Negatives and positives in the bristles assay are indicated with orange and green shading respectively.
(0.04 MB XLS)

Table S3 Results of the prioritization (Dec 2005) of the 12 positive deficiency regions, in 12 sheets, indicating the mutant alleles tested in each region and the result of the bristle assay. Negatives and positives are indicated with orange and green shading respectively.
(0.16 MB XLS)

Table S4 Results of the prioritization (June 2007) of the 12 positive deficiency regions, in 12 sheets, indicating the RNAi lines tested in each region and the result of the bristle assay. Negatives and positives are indicated with orange and green shading respectively.
(0.58 MB XLS)

Text S1 Supplementary Analysis: comparison of HIGHFLY with existing tools through post-analysis.
(0.12 MB PDF)
