# Supplementary figures and images for: Integrating Computational Biology and Forward Genetics in Drosophila
Source: PLoS Genet. 2009 Jan 23;5(1):e1000351. doi: 10.1371/journal.pgen.1000351 (PMC2628282; doi:10.1371/journal.pgen.1000351)

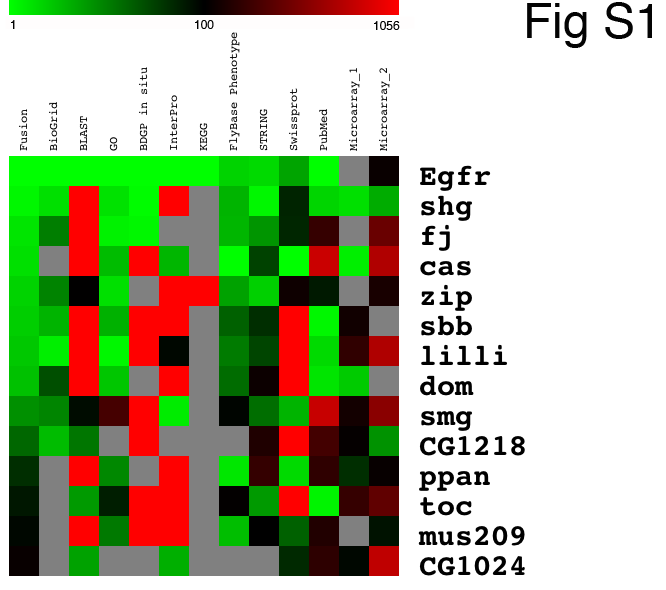

Supplement: Figure S1 — Contributions of the HIGHFLY data sources to the overall ranking. One HIGHFLY prioritization was performed on all 1056 genes that are contained within the 12 positive ato-specific deficiency regions. The first column shows the rank of the positive genes for the overall ranking obtained by the fusion of all the individual sources (columns 2–13). Grey squares represent missing data for that particular gene and data source. The genes with no or limited existing knowledge, such as CG1218 and CG1024 can still be ranked high. CG1218 is ranked high because of similarities with the training set through BioGRID (CG1218 interacts with sine oculis), BLAST (CG1218 has sequence similarity with chn, E-value 18.2) and Microarray_2 (similarities between CG1218 and the training set according to microarray gene expression data). CG1024 has similarities with the training set through BLAST (CG1024 has sequence similarity with senseless, E-value 0.54), InterPro (CG1024 contains a Zinc finger motif, C2H2-type, like senseless), and Swissprot (CG1024 contains the keyword “Zinc-finger, DNA binding”). (0.51 MB TIF) [file pgen.1000351.s001.tif]

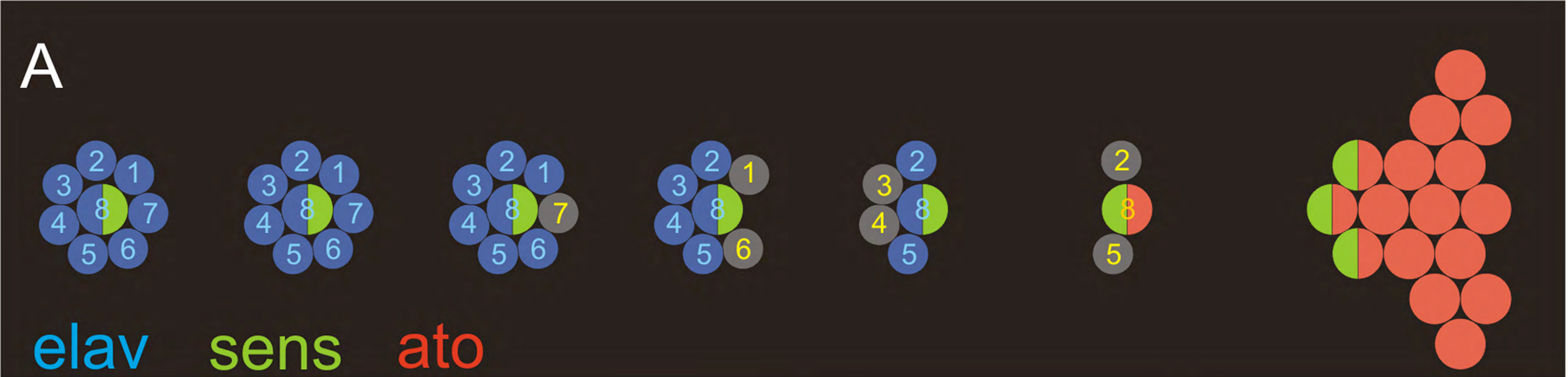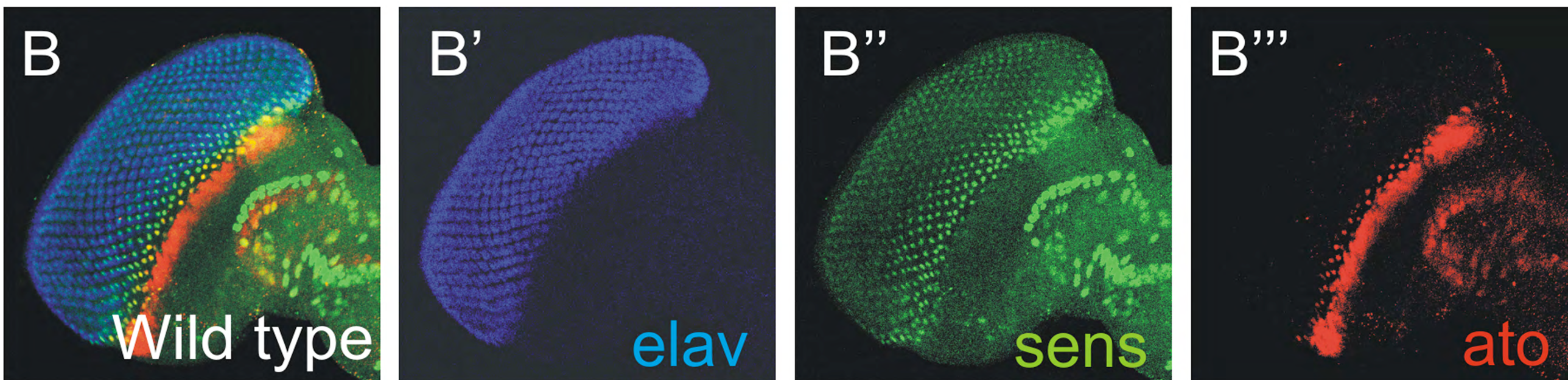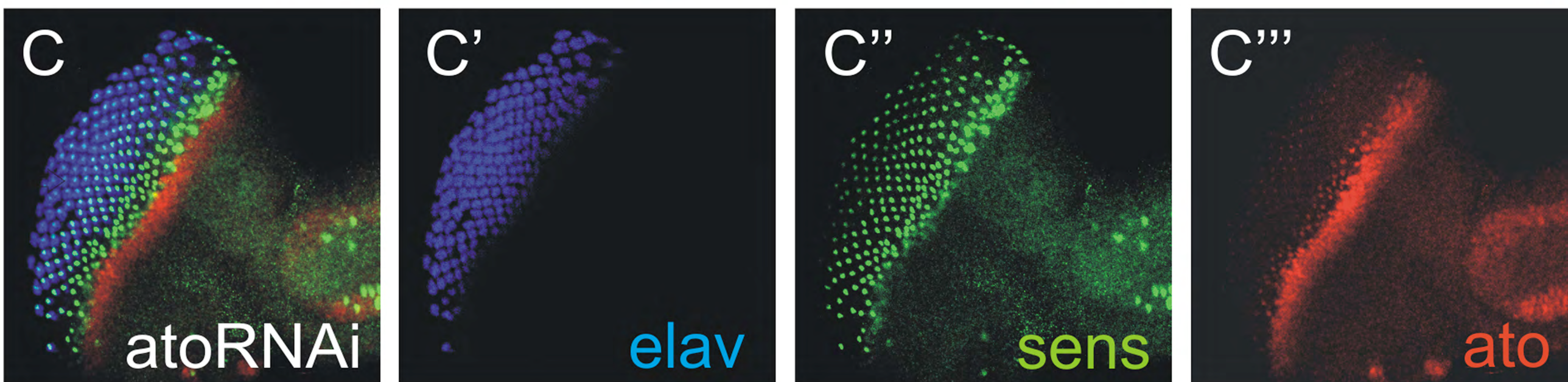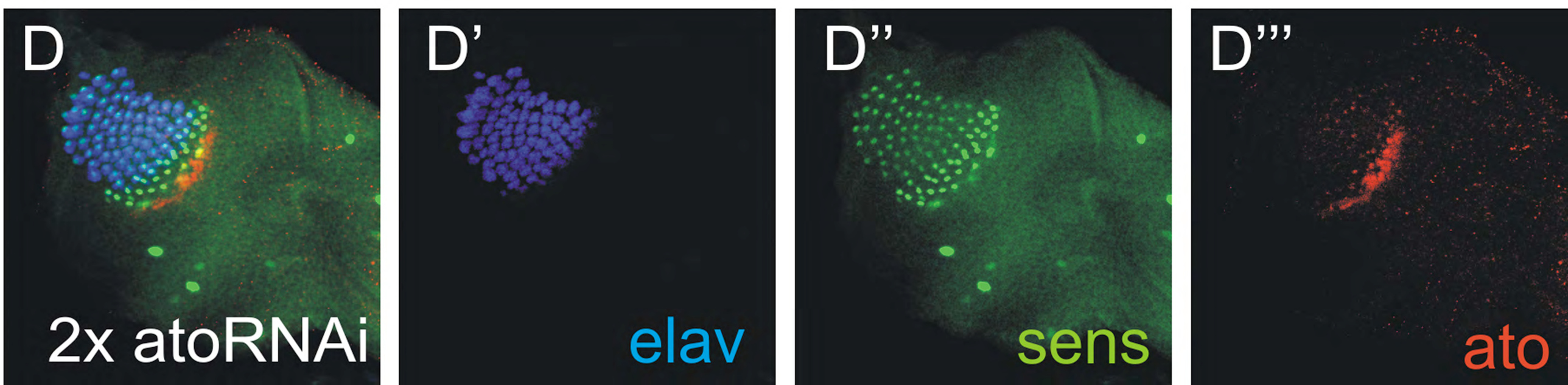

Supplement: Figure S2 — Expression of ato-RNAi inhibits retinal differentiation. Eye discs are oriented with posterior located to the left. A) Scheme of ato dependant retinal induction using the formation of one photoreceptor cluster as example, first Ato is expressed in a stripe of cells, than, due to lateral inhibition ato expressing cells are restricted to three cells and then a single cell, the R8, which begin to express Sens. The other 7 photoreceptors are recruited in a reiterative way. When these neurons mature they express Elav. B) wild type control eye disc stained for Elav (blue), Sens (green) and Ato (red). C, D) Expression of ato-RNAi causes dose-dependent loss of retinal differentiation with one copy (C) leading to the appearance of gaps in the Elav pattern, and two copies (D) leading to a major failure of photoreceptor differentiation. (0.93 MB PDF) [file pgen.1000351.s002.pdf]

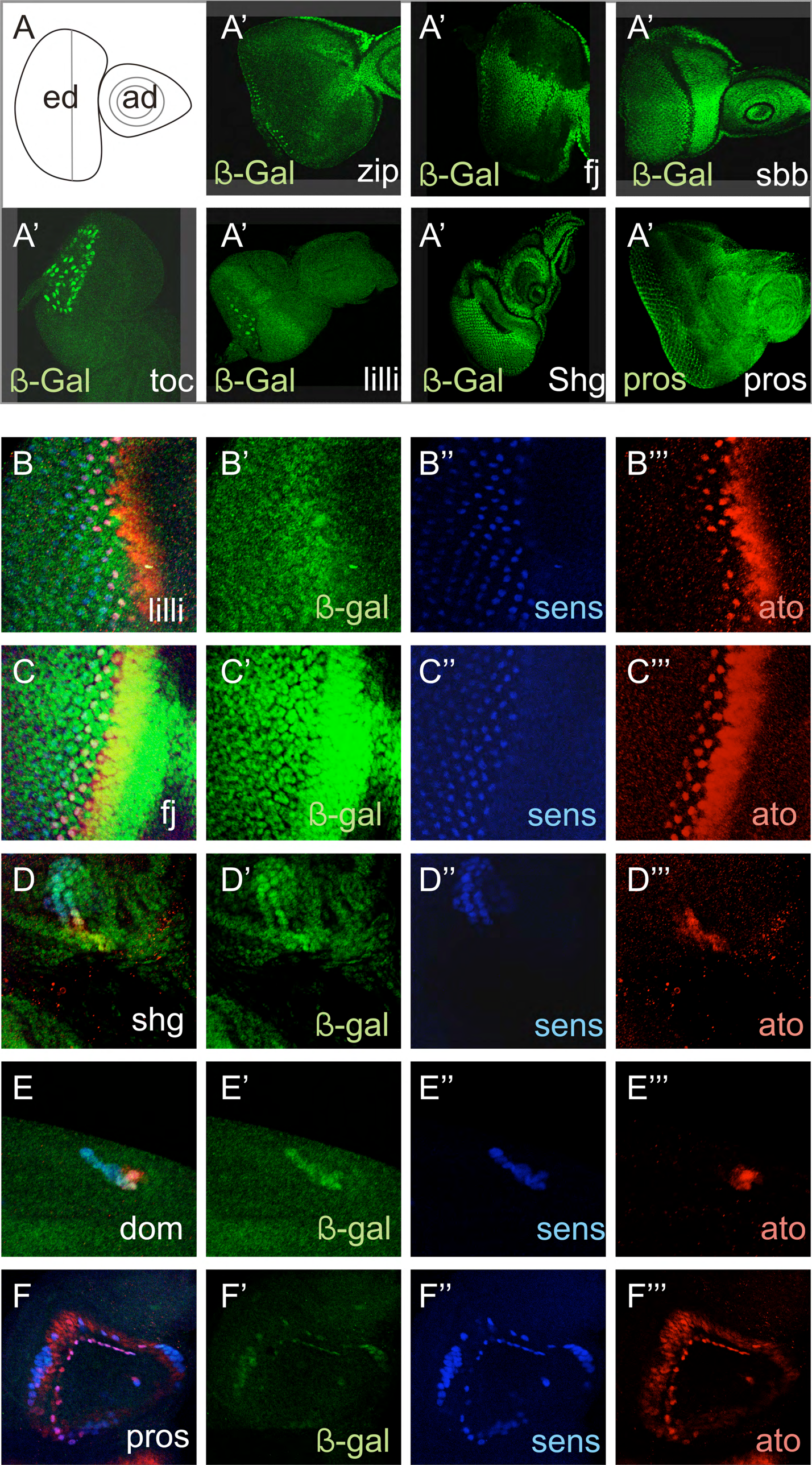

Supplement: Figure S3 — Overview of the expression pattern of ato interacting genes detected using LacZ enhancer trap lines or antibodies. (A) Overview of eye-antenna imaginal disc, with posterior to the right, ed: eye disc, ad: antenna disc. (A′) β-gal staining of LacZ enhancer trap flies mimicking the expression pattern of the genes nearby. Enhancer traps of zip, fj, sbb, shg, toc, and lilli show expression patterns in the eye disc. (B) eye disc of lilli enhancer trap flies, showing co-localization between Ato (B″) Sens (B′) and β-gal (B′). (C) Eye disc of fj enhancer trap flies, showing co-localization between Ato (C″) Sens (C′) and β-gal (C′). (D) Leg disc of shg enhancer trap flies, showing co-localization between Ato (D″) Sens (D′) and β-gal (D′) in the leg chordotonal organ precursor. (E) Wing disc of dom enhancer trap flies, showing co-localization between Ato (E″) Sens (E′) and β-gal (E′) in the wing chordotonal organ precursor. (F) Antennal disc of dom enhancer trap flies, showing co-localization between Ato (F″) Sens (F′) and β-gal (F′) in the Johnston organ precursor. (3.02 MB PDF) [file pgen.1000351.s003.pdf]
