# Supplementary material for: Integrating Computational Biology and Forward Genetics in Drosophila
Source: PLoS Genet. 2009 Jan 23;5(1):e1000351. doi: 10.1371/journal.pgen.1000351 (PMC2628282; doi:10.1371/journal.pgen.1000351)
Supplement: Text S1 — Supplementary Analysis: comparison of HIGHFLY with existing tools through post-analysis. (0.12 MB PDF) [file pgen.1000351.s008.pdf]

Supplementary Analysis: comparison of HIGHFLY with existing tools through post-analysis

Having found 16 positive genes that interact specifically with Atonal in various assays, we examined to what extent we could have found these positives through existing online databases. ENDEAVOUR-HIGHFLY integrates existing data sources, many of which are available online as individual databases that can be queried. The main differences with existing web applications are twofold. The first difference is that we use a set of training genes instead of a single query gene. This allows to "bias" the query towards a specific function of the query gene. For example, our query gene, *ato*, like many other genes, is actually involved in different developmental processes. Atonal can have both a proneural role (e.g., in eye and chordotonal organ development) and a neuronal differentiation role (in the brain). By using a set of training genes that consist of Atonal and 11 genes closely related to Atonal's proneural function (e.g., known to interact with Atonal; transcriptional target of Atonal; same pathway as Atonal; etc), we were able to positively bias our candidate set, resulting in a higher ranking of Atonal-related genes that are involved in Ato's proneural action. Another advantage of a training set is that the query becomes more sensitive (and less specific) so that not only are the known links with a query gene retrieved, but also new candidates can be predicted. Indeed, because we aim to identify novel interactors in a genetic screen, we choose to have a high sensitivity rather than a high specificity. A high specificity would only recover the genes that are already known to interact with Atonal. The second difference with other tools is that we combine multiple data sources through order statistics (i.e., integrate rankings across data sources), which alleviates any normalization procedure across different scoring functions.
We have chosen three websites (FlyBase, UCSC Gene Sorter, and STRING) to examine whether a "simple" analysis would have yielded the same positives and whether fewer or more candidates would be predicted to be tested *in vivo*. These tools are all very easy to use and are extremely valuable for particular goals. However, they all lack the possibility to use a training set of genes; and all except STRING do not allow combining multiple data sources; while we believe that these are key features that allow for a strong improvement of candidate gene selection for a medium-throughput genetic assay.

FlyBase
The first tool is FlyBase[1] itself, from which HIGHFLY uses a number of data sources, namely Gene Ontology (GO) and phenotypes. FlyBase offers a QueryBuilder tool that allows retrieving all genes using an expert-chosen query. We used QueryBuilder to retrieve all genes that are annotated with "relevant" GO terms for our process under study. Relevant terms were chosen based on the current GO annotation of Atonal itself. A second type of query we performed with QueryBuilder was to retrieve all genes that are known to be expressed in "relevant tissues" for our process. Again, relevant tissues were decided based on the tissues where Atonal is known to be expressed (given in FlyBase's "Gene Expression Report"). These types of queries result in a list ("bag") of genes, but this list is not ranked according so similarity. This means that all candidates have to be tested in the genetic assay. This makes this procedure less suited for candidate gene selection for knowledge-guided genetic screens when the query yields too few or too many candidates.

Here is the query we used for GO:

```
# Query data for session 19497
target=fbgn
species=Dmel
guistyle=1

OR	fbgn	fbgn-GO_BIOLOGICAL_PROCESS	GO biological process	acc	no
OR	fbgn	fbgn-GO_BIOLOGICAL_PROCESS	GO biological process	GO:0000187	no
OR	fbgn	fbgn-GO_BIOLOGICAL_PROCESS	GO biological process	GO:0007460	no
OR	fbgn	fbgn-GO_BIOLOGICAL_PROCESS	GO biological process	GO:0007605	no
OR	fbgn	fbgn-GO_BIOLOGICAL_PROCESS	GO biological process	GO:0016330	no
OR	fbgn	fbgn-GO_BIOLOGICAL_PROCESS	GO biological process	GO:0016360	no
OR	fbgn	fbgn-GO_BIOLOGICAL_PROCESS	GO biological process	GO:0045165	no
OR	fbgn	fbgn-GO_BIOLOGICAL_PROCESS	GO biological process	GO:0045464	no
OR	fbgn	fbgn-GO_BIOLOGICAL_PROCESS	GO biological process	GO:0045465	no
OR	fbgn	fbgn-GO_BIOLOGICAL_PROCESS	GO biological process	GO:0048800	no
OR	fbgn	fbgn-GO_BIOLOGICAL_PROCESS	GO biological process	GO:0007455	no
OR	fbgn	fbgn-GO_BIOLOGICAL_PROCESS	GO biological process	GO:0001745	no
OR	fbgn	fbgn-GO_BIOLOGICAL_PROCESS	GO biological process	GO:0001746	no
OR	fbgn	fbgn-GO_BIOLOGICAL_PROCESS	GO biological process	GO:0001748	no
OR	fbgn	fbgn-GO_BIOLOGICAL_PROCESS	GO biological process	GO:0007173	no
OR	fbgn	fbgn-GO_BIOLOGICAL_PROCESS	GO biological process	GO:0007224	no
OR	fbgn	fbgn-GO_BIOLOGICAL_PROCESS	GO biological process	GO:0007423	no
OR	fbgn	fbgn-GO_BIOLOGICAL_PROCESS	GO biological process	GO:0007422	no
```

And this is the query used for gene expression:

```
# Query data for session 11238
target=fbgn
```

```
species=Dmel
guistyle=1
OR	fbgn	fbgn-POLYPEPTIDE_EXPRESSION_DATA	polypeptide expression data	chordotonal	no
OR	fbgn	fbgn-POLYPEPTIDE_EXPRESSION_DATA	polypeptide expression data	photoreceptor	no
OR	fbgn	fbgn-POLYPEPTIDE_EXPRESSION_DATA	polypeptide expression data	eye-antennal	no
OR	fbgn	fbgn-POLYPEPTIDE_EXPRESSION_DATA	polypeptide expression data	morphogenetic furrowno
OR	fbgn	fbgn-POLYPEPTIDE_EXPRESSION_DATA	polypeptide expression data	inner proliferation zone	no
OR	fbgn	fbgn-POLYPEPTIDE_EXPRESSION_DATA	polypeptide expression data	Johnston	no
OR	fbgn	fbgn-TRANSCRIPT_EXPRESSION_DATA	transcript expression data	chordotonal	no
OR	fbgn	fbgn-TRANSCRIPT_EXPRESSION_DATA	transcript expression data	photoreceptor	no
OR	fbgn	fbgn-TRANSCRIPT_EXPRESSION_DATA	polypeptide expression data	eye-antennal	no
OR	fbgn	fbgn-TRANSCRIPT_EXPRESSION_DATA	polypeptide expression data	morphogenetic furrowno
OR	fbgn	fbgn-TRANSCRIPT_EXPRESSION_DATA	polypeptide expression data	inner proliferation zone	no
OR	fbgn	fbgn-TRANSCRIPT_EXPRESSION_DATA	polypeptide expression data	Johnston	no
```

Note: FlyMine[2] is another useful web application that makes FlyBase data and other functional genomics data available. However, we did not include FlyMine in this analysis because the FlyMine project has unfortunately announced it will no longer be updated after December 2008. FlyMine allows building similar queries like we performed with FlyBase QueryBuilder, and allows for several more genomic data sources to be used in the query. However, HIGHFLY's main advantages, like the use of training sets and the generations of combined rankings, are not available in FlyMine.

UCSC Gene Sorter

The second tool we used was UCSC Gene Sorter[3]. This very efficient tool ranks all genes in the genome (for which data is available in the chosen data source) according to one chosen data source and one query gene. The ranked list can also be filtered. In our case we used all genes in our positive deficiency regions as filter. Many of the data sources in the Gene Sorter are the same as we use in ENDEAVOUR-HIGHFLY (e.g., GO, gene expression from microarray data, protein-protein interactions, protein sequence similarity, protein domain similarity). We have chosen three data sources as illustration, namely GO, expression, and protein-protein interactions. An important difference with FlyBase QueryBuilder, when using GO, is that Gene Sorter calculates a GO similarity, and not only retrieves genes that are annotated with the same GO term. Therefore, this tool is more suited for candidate gene selection for genetic screens. However, as already mentioned, this tool does not allow to combine the different data sources into a single fused ranking, nor does it allow to use a set of training genes as query.

STRING

The last tool we used was STRING[4]. This tool shares an important feature with our method, namely the integration of data from various heterogeneous sources, both experimental data (e.g., gene expression, protein-protein interactions), and derived data (e.g., text-mining). STRING can be used to detect known and predicted associations with a query gene or a list of query genes. The results are presented as a network, which can be saved as text file, together with their confidence scores. This way, one can retrieve a ranked list (based on the confidence score) of predicted associations. In the first analysis we used "Atonal" as query gene and retrieved all 228 predicted associations. Unfortunately, STRING does not allow a filter on the genome, so we compared these 228 offline with our candidate set of 1056 genes from our positive deficiency regions. Also, to circumvent STRING's automatic mapping of gene identifiers (we used CG gene identifiers as input), we downloaded the fasta file, which also contains the CG number. We found an overlap of 13 genes, of which 2 were positive in our genetic assay.

In a second analysis we used STRING's multiple gene input function. Note that the input of multiple genes may resemble our use of a training set, but an important difference is that STRING returns individual interactions with and among the input genes, while HIGHFLY integrates the training genes to build a summarized data models across that training set. To compare these two approaches, we used the same training set as multiple gene input in STRING. Unfortunately, the maximum number of allowed interactions in STRING is 500. Using this threshold, we retrieved 500 associations with the genes in our training set, of which 35 fall into our positive deficiency regions, and of which 5 are positive Ato-interactors.

Existing tool	Ref	Goal	Query	Result	# Genes to test from positive deficiencies	# Positive genes recovered[d]
**ENDEAVOUR-HIGHFLY**	This study	Prioritize list of "test genes" based on set of "training genes"	12 training genes related to Atonal proneural function	Prioritized genes from the deficiency regions[a]	Start with highest-ranking genes	12 in top 100<br>all 14 in top 200
**FlyBase QueryBuilder[b] (FB2008_08)**	[1]	Retrieval of genes based on user-defined query terms	Ato-related **GO** terms[c] combined with "OR"	449 genes	449 genes (no ranking)	2 (Egfr, shg)
			Ato-related **expression**	210 genes	210 genes (no ranking)	3 (Egfr, fj, sbb)

			**patterns** combined with “OR”			
			GO and expression combined with “OR”	591 genes	591 genes (no ranking)	4 (Egfr, fj, shg, sbb)
**UCSC Gene Sorter (April 2006 Assembly)**	[3]	Prioritize whole genome (or filtered genome) based on a query gene	“ato” + filter “paste list” of all genes in deficiency regions; “GO similarity”	Prioritized genes from deficiency regions	Start with highest-ranking genes	6 in top 100<br>all 14 in top 668
			Idem for “expression similarity”	idem	Idem	3 in top 100 (fj, smg, shg)<br>all 14 in top 697
			Idem for “protein-protein interactions”	idem	idem	5 in top 100<br>all 14 in top 702
**STRING version 8.0 Preview**	[4]	Predict associations with a query gene, based on experiments, text-mining, and other data sources	“ato”	Ranked list of predicted associations with ato and its ‘neighborhood’	Among the 228 predicted associations, 13 genes overlap with our test set	2 from 13 (Egfr and lilli)
			Same 12	Associations	By setting the maximal	5 from 35

			training genes as used in HighFly using the "multiple gene names" input function of STRING	among the input list	number of interactors to the maximum allowed (500), 35 genes overlap with our test set	

[a] for this analysis we have grouped all 1056 genes that are found within the 12 positive deficiency regions into one test set, to compare the results of one analysis instead of calculating statistics on the results of 12 separate analyses.
[b] FlyBase QueryBuilder queries can be found as supplementary data; these can be uploaded in FlyBase QueryBuilder.
[c] GO annotations of Atonal, removing: component terms (nucleus), function terms (transcription factor), and CNS-related process terms.
[d] The set of 14 positive genes consist of 12 positive 'known' genes from our deficiency screen (*cas, dom, Egfr, fj, lilli, mus209, ppan, sbb, shg, smg, toc,* and *zip*) and two 'unknown' genes from our RNAi screen (CG1024 and CG1218).

References

1. Tweedie S, Ashburner M, Falls K, Leyland P, McQuilton P, et al. (2008) FlyBase: enhancing Drosophila Gene Ontology annotations. Nucleic Acids Res.
2. Lyne R, Smith R, Rutherford K, Wakeling M, Varley A, et al. (2007) FlyMine: an integrated database for Drosophila and Anopheles genomics. Genome Biol 8: R129.
3. Hinrichs AS, Karolchik D, Baertsch R, Barber GP, Bejerano G, et al. (2006) The UCSC Genome Browser Database: update 2006. Nucleic Acids Res 34: D590-598.
4. von Mering C, Jensen LJ, Kuhn M, Chaffron S, Doerks T, et al. (2007) STRING 7--recent developments in the integration and prediction of protein interactions. Nucleic Acids Res 35: D358-362.
